# Serologic Evidence of Hantavirus Infection in Humans, Colombia

**DOI:** 10.3201/eid1012.040821

**Published:** 2004-12

**Authors:** Salim Máttar, Miguel Parra

**Affiliations:** * Universidad de Córdoba, Montería, Colombia;; † Corporación Universitaria del Sinú, Montería, Córdoba, Colombia

**Keywords:** letter, hantavirus, Colombia, South America

**To the Editor:** Several New World hantaviruses cause hantavirus pulmonary syndrome (HPS) in the Americas. All hantaviruses that cause HPS are hosted by the rodent family *Muridae*, subfamily *Sigmodontinae* (New World rats and mice). Since the Sin Nombre virus (SNV) was documented in 1993 (*1*), ≈25 sigmodontine hantavirus genotypes from the Americas have been described; each is associated with a different rodent species or subspecies. Hantaviruses have been documented in South America from Argentina, Chile, Paraguay, Uruguay, Bolivia, Brazil, Peru, and Venezuela (*2*), and in Central America from Costa Rica and Panama (*3**,**4*).

Although documented in four bordering countries, hantaviruses have not been documented in Colombia. We assessed hantavirus antibody prevalence in humans by screening blood samples from rural volunteers within Córdoba and Sucre provinces in Colombia. Workers 16–65 years-of-age from 12 communities were enrolled. The research committee of the University of Córdoba approved the protocol, and informed consent was obtained from all participants. Participants were of low socioeconomic status and lived in homes with no running water and frequently no electricity. These workers had lived in the same general area all of their lives; none had traveled outside of Colombia. From January to October 2003, 88 blood samples were collected in 12 localities. Samples were screened for immunoglobulin (Ig) G antibody reactive with SNV antigen by using enzyme immunoassay (*5*). This assay detects, but does not distinguish among, all known sigmodontine hantaviruses. SNV antibody was detected in 12 samples (13.5%) representing 10 of 12 sites. Site-specific prevalences (Figure) ranged from 5% (1 of 19) to 50% (1 of 2). Except for one category with no positive samples, prevalence of anti-SNV IgG was similar across occupations (chi square = 0.03, df = 3, p = 0.998). All 12 antibody-positive samples were from male workers. We divided the study population into five age categories (18–24 years, n = 19; 25–34 years, n = 24; 35–44 years, n = 20; 45–54 years, n = 15; 55–70 years, n = 10) and found significant differences among the proportions of antibody-positive persons (chi square = 9.8, df = 4, p = 0.04). The higher prevalences were in the two youngest age groups (16%–17%) and the oldest age group (40%); one antibody-positive sample was found in the 35- to 54-year-old group (3%).

**Figure:**
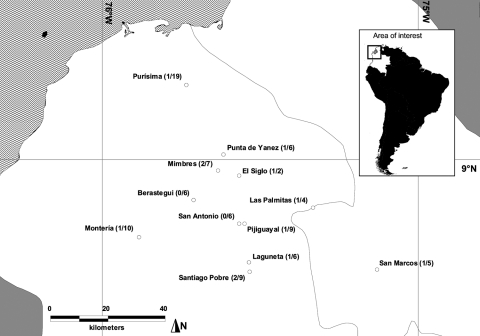
Locations of 12 towns in Córdoba and Sucre departments, Colombia, where rural workers were screened for antibody to Sin Nombre hantavirus. Numbers in parentheses represent number of antibody-positive persons and number of persons tested.

The prevalence of SNV-reactive antibody in rural workers indicates that at least one hantavirus is endemic in rodents in northern Colombia and is frequently transmitted to rural residents. This finding further supports mounting evidence that hantaviruses and HPS are a Pan-American problem (*2**,**6*). The bimodal infection distribution among age groups suggests human exposure might be episodic with an extended periodicity. Preliminary and limited rodent sampling has not produced any hantavirus antibody–positive samples. Although their distributions are poorly studied, both *Zygodontomys brevicauda* (reservoir of Calabazo virus in Panama) and *Oligoryzomys fulvescens* (reservoir of Choclo virus, a known agent of HPS in Panama [*4*]) are believed to be found in northern Colombia (*7*).

Despite the prevalence of antibodies to a hantavirus in humans in northern Colombia, we did not find any human illnesses. None of the SNV antibody–positive volunteers reported illness compatible with HPS; however, some hantaviruses may cause mild or no illness. Surveys have shown hantavirus antibodies (13%–40% prevalence in Paraguay and northern Argentina [*8*], Bolivia [J. Montgomery et al., unpub. data], northern Brazil [P.F.C. Vasconcelos et al., unpub. data], and Panama [*6*]) in some populations with little evidence of illness.

Infection in rural workers and the likely presence of *O. fulvescens* in northern Colombia underscore the importance of physician awareness and surveillance for HPS. A systematic survey of sigmodontine rodent populations that will identify the hantaviruses and their hosts is imperative.
